# Oxidative Stress and Alterations of Paraoxonases in Atopic Dermatitis

**DOI:** 10.3390/antiox10050697

**Published:** 2021-04-28

**Authors:** Oriana Simonetti, Tiziana Bacchetti, Gianna Ferretti, Elisa Molinelli, Giulio Rizzetto, Luisa Bellachioma, Annamaria Offidani

**Affiliations:** 1 Department of Clinical and Molecular Sciences-Dermatology, Polytechnic University of Marche, I-60126 Ancona, Italy; molinelli.elisa@gmail.com (E.M.); grizzetto92@hotmail.com (G.R.); a.offidani@ospedaliriuniti.marche.it (A.O.); 2 Department of Life and Environmental Sciences-Biochemistry, Polytechnic University of Marche, I-60126 Ancona, Italy; t.bacchetti@staff.univpm.it (T.B.); luisabellachioma@gmail.com (L.B.); 3 Department of Clinical Experimental Science and Odontostomatology-Biochemistry, Research Center of Health Education and Health Promotion, Polytechnic University of Marche, I-60126 Ancona, Italy

**Keywords:** atopic dermatitis, myeloperoxidase, paraoxonases, oxidative stress

## Abstract

Background: previous studies reported the involvement of reactive oxygen species (ROS) and lipid peroxidation in the pathogenesis of inflammatory skin diseases. The aim of our study was to investigate the relationship between oxidative stress and inflammation in children affected by atopic dermatitis (AD), a chronic relapsing inflammatory skin disease. Methods: levels of lipid hydroperoxides, total antioxidant capacity, and activities of the enzymes myeloperoxidase (MPO), PON1, and PON2/3 were investigated in 56 atopic pediatric patients, and compared with 48 sex-/age-matched healthy controls. Results: significantly higher levels of lipid hydroperoxides and lower values of total antioxidant potential were observed in the serum of AD children compared to that of the controls. Significant lower PON1 activities, and a significant increase in levels of MPO were observed in serum of patients, with a higher serum MPO level/PON1 paraoxonase activity ratio in patients compared to that in the controls. Significantly lower lactonase activity of PON enzymes was observed in polymorphonuclear cells isolated from AD patients. Statistically negative correlation was established between the activity of intracellular PON2/3 activity and ROS levels. Conclusions: our data confirmed that AD is associated with higher oxidative damage and a decrease in antioxidant defense. Moreover, alterations of extracellular and intracellular PON activity can promote lipoprotein dysfunction in AD patients.

## 1. Introduction

Atopic dermatitis (AD) is a chronic relapsing inflammatory skin disease affecting up to 25% of children, with age and ethnic differences [1]. Its clinical manifestations include itching and scratching, dry skin, patchy eczema (especially on flexural locations), exudation, and skin thickening and discoloration. Although the pathogenesis of AD is not completely understood, genetic, environmental, and/or psychological triggers appear to be involved in and contribute to the infiltration of inflammatory cells, such as lymphocytes, macrophages, eosinophils, and mast cells [2]. The chronically inflamed skin of patients with atopic dermatitis plays a key role in the pathogenesis of this disease, with the overproduction of reactive oxygen species (ROS) [3,4,5,6]. The overproduction of ROS was observed in skin biopsies in AD patients compared to controls [6]. Studies in children and adults demonstrated higher levels of biomarkers of oxidative stress of lipids and nucleic acids in serum and urine [3]. Alterations of serum NO levels, malondialdehyde (MDA), and 8-hydroxydeoxyguanosine (8-OHdG) were described [5,7,8]. However, contrasting results were reported by other authors [9]. Among antioxidant enzymes, previous studies showed a decrease in superoxide dismutase (SOD), catalase, and glutathione peroxidase (GPX) in patients compared to controls [10]. In addition, the levels of nonenzymatic antioxidant parameters such as GSH, and vitamins A, E, and C also decreased in patients compared to controls [10,11]. 

The increased generation of reactive oxygen species (ROS) and alterations of antioxidant enzymes are involved in the pathogenesis of several chronic diseases [12]. Oxidative damage in vivo is described as an imbalance between free radicals (ROS) that acutely or chronically exceed antioxidant defense capacity. In excess, oxidants can react with all cellular macromolecules, including lipids, proteins, and nucleic acids. Increased ROS production originates from endogenous sources, such as the inflammatory responses of leucocytes involving NADPH-oxidase (NOX), inducible nitric oxide synthase (iNOS), and myeloperoxidase (MPO). Of the circulating MPO, 95% is derived from neutrophils, and to a lesser extent from monocytes [13]. A major biological function of MPO is the defense of the organism against infections by generating antimicrobial oxidants, free radicals, and other reactive oxygen species [13]; however, MPO activity can also lead to oxidative damage of the endothelium, and promote the oxidation of plasma lipids and lipoproteins LDL [14] and HDL [15,16,17]. In fact, activated neutrophils lead to the production of ROS, such as hydrogen peroxide (H_2_O_2_), hydroxyl radical, superoxide radical, and nitrogen-containing radicals, such as peroxynitrite and hypochlorous acid (HOCl) from H_2_O_2_ via the myeloperoxidase enzyme [13]. Recently, MPO was found to be implicated in several human diseases associated with oxidative stress, such as atherosclerosis [18] and psoriasis [19]. A recent proteomic study showed the upregulation of key proinflammatory proteins, including MPO, in the serum of AD patients [20].

The aim of our study was to investigate the relationship between oxidative stress and inflammation in AD children. Therefore, the comparison of lipid hydroperoxides (LOOHs) levels as biochemical markers of serum lipoprotein peroxidation was associated with the study of total antioxidant capacity and of the activities of enzymes MPO and paraoxonase 1 (PON1) in the serum of patients and controls. Moreover, we studied the activity of paraoxonase 2/3 (PON2/3) in the peripheral blood mononuclear cells (PBMNCs) of controls and AD patients. The purpose of our study is supported by previous research that showed that enzymes of the PON family exert a key role against inflammation and oxidative stress [21]. PON1 is associated with high-density lipoprotein (HDL) [22,23,24,25]; PON2 and PON3 are intracellular enzymes, and they exert antioxidant roles in mitochondria and plasma membrane [26,27,28,29]. PON1 was widely investigated in cardiovascular disease and other human diseases associated with oxidative stress [22,23,24,25]; PON2 and PON3 were less thoroughly investigated.

Among factors involved in the oxidative stress of plasma lipoproteins, increasing attention is devoted to the MPO enzyme, and a relationship between MPO and PON1 activity was proposed [15,16,17]. The ratio between the serum levels of MPO and PON1 activity is described as a potential indicator of dysfunctional HDL [15,30]. A high serum MPO/PON1 ratio is described in patients affected by inflammatory diseases [15,19], but it was not previously studied in AD.

## 2. Materials and Methods

### 2.1. Subjects 

For this study, 56 patients (31 males, 26 females) aged 18 months to 12 years, who had been diagnosed with chronic AD, were recruited from the Clinic of Dermatology of Ancona (Ancona, Italy).

All subjects were diagnosed with AD by pediatric dermatologists according to the criteria developed by Hanifin and Rajka [31]. Clinical severity was determined using the scoring of atopic dermatitis (SCORAD) index, and classified as mild (<15), moderate (15–40), or severe (≥40) [32]. Subjects with mild-to-moderate AD (SCORAD 15–40) were included.

The following parameters were evaluated: age, sex, and body-mass index. The presence of clinical signs, including atopy, asthma, and rhinitis, was also recorded in the AD group (as illustrated in Table 1).

AD patients under treatment with systemic steroid or immune suppressant, or subjects with diabetes, clinical evidence of cardiovascular diseases, or who had been receiving lipid-lowering drugs or antioxidant supplements were excluded from the study to avoid possible interferences on PON1 activity and plasma lipids.

A total of 48 healthy subjects (26/22 F/M), age- and sex-matched, without skin or systemic inflammatory disease and without personal or family history of atopic disease, including AD, bronchial asthma, or allergic rhinitis, were also recruited. 

The study was conducted in accordance with the provisions of the Declaration of Helsinki, the International Conference on Harmonization Good Clinical Practices guideline, and applicable regulatory requirements. The study was approved by the Ethics Committee of Ospedali Riuniti di Ancona. Written informed consent was obtained for all patients from a parent or legal guardian.

### 2.2. Sample Collection

Blood samples of controls and AD children were collected at 8 a.m. after overnight fasting. Venous blood samples (about 10 mL) were collected in two vacuum tubes. An aliquot (about 5 mL) was used for serum separation; the tube containing blood without anticoagulant was left at room temperature for 30 min to allow for clot formation. The clot was removed by centrifuging at 1000–2000× *g* for 10 min in a refrigerated centrifuge. Serum samples were divided in aliquots and stored immediately at −80 °C. An aliquot of blood (5 mL) was incubated with EDTA and then immediately used for isolation of PBMNCs.

### 2.3. Routine Blood Tests

Fasting levels of total cholesterol (TC), HDL-cholesterol (HDL-C), LDL-cholesterol (LDL-C), and triglycerides (TG) were measured using standard methods. Apolipoprotein AI was measured by a turbidimetric latex agglutination assay. High-sensitivity C-reactive protein (hs-CRP) level was measured by a nephelometric assay.

### 2.4. Biomarkers of Lipid Peroxidation

The levels of lipid hydroperoxides were determined in serum using a ferrous oxidation−xylenol orange (FOX2) assay as previously described [19]. The levels of lipid hydroperoxides were quantified using a stock solution of t-butyl hydroperoxide. Results are shown as µmol of lipid hydroperoxides for L of serum.

### 2.5. Serum Total Antioxidant Capacity

An oxygen radical absorbance capacity (ORAC) assay was used to evaluate total antioxidant capacity in serum [33]. The ORAC assay was conducted at 37 °C using fluorescein as the substrate, and 2,2-azobis (2-amidinopropane) dihydrochloride (AAPH) as the free radical initiator. Serum was diluted with 75 mM phosphate buffered saline (PBS) in 1:250 ratio. Standard or diluted samples of 25 µL and 150 µL of fluorescein (0.08 μM) were mixed in 96 well black plates. In the blank samples, 75 mM PBS was added. Trolox was used as standard. Reaction mixtures were incubated at 37 °C in a microplate reader for 30 min prior to the addition of 25 µL of AAPH (150 mM). The plate was placed in the fluorescence microplate reader, and kinetic reads were performed at 5 min intervals for 2 h at an excitation wavelength of 485 nm and an emission wavelength of 525 nm. Fluorescein-decay curves were based on individual well fluorescence relative to the initial value and derived net area under each curve (AUC). The AUC of the standards were used to derive the standard curve. Data are expressed as antioxidant activity in Trolox equivalents on the basis of the above-derived standard curve. Results are expressed as mmol Trolox equivalents (TE)/L (mmol TE/L). 

### 2.6. Isolation of PBMNCs

Blood samples containing EDTA were used for the isolation of PBMNCs by density centrifugation on Ficoll Paque™ Plus (1.0077 g/mL) at 630× *g* for 30 min. Cell viability by Trypan Blue exclusion was ≥90%.

### 2.7. Detection of Intracellular ROS in PBMNCs

Intracellular ROS levels were evaluated in PBMNC using 2,7-dichlorofluorescin-diacetate (DCFH-DA) (Sigma–Aldrich, St. Louis, MO, USA). DCFH-DA is readily taken up by cells and subsequently converted to 2′,7′-dichlorodihydrofluorescein (DCFH), which can be oxidized to dichlorofluorescein (DCF) by hydrogen peroxide, peroxynitrite, and other intracellular ROS or reactive nitrogen species. Cells (1 × 10^6^/mL) were pretreated for 45 min at 37 °C with DCFH-DA (10 μM) in the dark. Cells were washed by centrifugation, resuspended in PBS, and then seeded in 96-well microplate. Fluorescence (Ex 480 nm; em 530 nm) was recorded in a microplate fluorescence reader from Bio-Tek Instruments, Inc (Winooski, VT, USA).

### 2.8. Serum Paraoxonase-1 Activities

PON1 activities were evaluated using three substrates: paraoxon for paraoxonase activity, phenylacetate for arylesterase activity, and dihydrocoumarin for lactonase [19]. All assays were performed in a 96-well plate in a total reaction volume of 200 µL. Each sample was run in triplicate wells, and the average value was used in the analyses; control samples were run on each plate. Intra- and interassay coefficients of variation were <3% in the tests.

#### 2.8.1. Paraoxonase Activity

Plasma of 10 µL in volume (non-diluted samples) was used. The basal assay mixture included 5 mmol/L Tris-HCl, pH 7.4, containing 1 mmol/L CaCl_2_ and 1.0 mmol/L paraoxon. Paraoxon hydrolysis was spectrophotometrically monitored for 8 min every 15 s at 412 nm. Nonenzymatic hydrolysis of paraoxon was subtracted from the total rate of hydrolysis. PON1 paraoxonase activity is reported as nmol of substrate hydrolyzed per minute per mL of undiluted serum.

#### 2.8.2. Arylesterase Activity

Plasma samples were diluted 1:10 with 1 mmol/L CaCl_2_ in 50 mmol/L TrisHCl, pH 8.0; then, 5 µL was taken for a total reaction volume of 200 µL. After the addition of the substrate phenyl acetate (1 mmol/L), the hydrolysis was monitored at 270 nm for 3 min (every 15 s). Nonenzymatic hydrolysis of phenyl acetate was subtracted from the total rate of hydrolysis. PON1 arylesterase activity is reported as U/mL; 1 U/mL is defined as 1 μmol of substrate hydrolyzed per minute per 1 mL of undiluted serum. 

#### 2.8.3. Lactonase Activity

Serum samples were diluted 1:10 with 1 mmol/L CaCl_2_ in 50 mmol/L TrisHCl, pH 8.0, and 3 µL was then taken for the assay. After the addition of the substrate dihydrocoumarin (DHC) (1.0 mmol/L), the hydrolysis was monitored at 270 nm for 10 min (every 15 s). Nonenzymatic hydrolysis of DHC was subtracted from the total rate of hydrolysis. PON1 lactonase activity is reported as U/mL; 1 U/mL is defined as 1 μmol of substrate hydrolyzed per minute per 1 mL of undiluted serum. 

### 2.9. Paraoxonase Activity in PBMNCs

PON2/3 activity in PBMNCs was evaluated using dihydrocoumarin (DHC) as substrate [34,35]. PBMNCs were suspended in Tris buffer (25 mmol/L Tris/HCL, pH 7.6, 1 mmol/L CaCl2) and sonicated on ice. Lysed PBMNCs protein concentration was determined using the Bradford method and then diluted to 1 mg protein/mL. The standard assay mixture contained 25 mM Tris-HCl, pH 7.6, 1 mM CaCl_2_, 1 mM DHC. Enzyme activities were measured using 200 μL of sonicate (200 μg protein) per mL assay mixture. The increase in rate absorbance was spectrophotometrically recorded at 270 nm. Nonenzymatic hydrolysis of DHC was subtracted from the total rate of hydrolysis. PON2/3 lactonase activity was expressed as U/mg protein; one unit of lactonase activity is equal to 1 μmol of DHC hydrolyzed per minute of mL of lysate, and was then converted into U/mg. 

In our conditions, each sample was run in triplicate wells, and the average value was used in the analyses; the CV for the triplicate variation was <4%.

To demonstrate that lactonase activity in PBMNCs is attributed to intracellular PONs, we used EDTA in preliminary experiments to chelate calcium ions that are required for the enzymatic activities of PONs. The addition of EDTA in sonicated PBMNCs resulted in a marked inhibition (92%) of lactonase activity evaluated using DHC. Using PON1 specific substrate, paraoxon, no paraoxonase activity was observed in PBMNCs, suggesting a lack of PON1 in these cells (data not shown). These results suggested that lactonase activity observed in PBMNCs is related to the DHC hydrolysis catalyzed by PON2 and PON3.

### 2.10. Serum Myeloperoxidase Levels 

A solid phase two-site MPO ELISA Kit from Mercodia (Uppsala, Sweden) was used to evaluate serum MPO. Serum samples of controls and patients were included on each plate. Results were reported as ng of MPO for mL of serum.

### 2.11. Statistical Analysis

Results are reported as mean ± standard error (SE). For the comparison of normally distributed variables between groups, the Student’s *t*-test was used. Paraoxonase activity showed a non-Gaussian distribution; therefore, we used a nonparametric test (Wilcoxon rank-sum test). Pearson’s correlation coefficients and their significance levels were calculated for linear-regression analysis. Differences were considered to be statistically significant at *p* < 0.05 (Microcal Origin 5.0, OriginLab, Northampton, MA, USA). 

## 3. Results

### 3.1. Clinical Data

The study of lipid profile in the serum of AD patients compared to the controls did not show significant differences. A lower level of ApoAI was observed in patients compared to that in the controls, but the difference was not significant. No significant modifications were observed in serum CRP levels (as illustrated in Table 1).

### 3.2. Biomarkers of Lipid Peroxidation 

As reported in Table 2, significantly higher levels of lipid hydroperoxides and lower values of total antioxidant potential were observed in AD children compared to healthy sex- or age-matched control children (*p* < 0.001). 

### 3.3. PON1 Activity and MPO Levels

In the serum of AD children, significantly lower activity of PON1 (paraoxonase, arylesterase, and lactonase) was observed compared to that in the controls (*p* < 0.001). Paraoxonase, arylesterase, and lactonase activities in AD were decreased by 39%, 28%, and 28%, respectively. In the same patients, a significant increase in serum levels of myeloperoxidase (MPO) was found (*p* < 0.001, Table 2). The comparison of the ratio between serum MPO level/PON1 paraoxonase activity (MPO/PON1 ratio) showed higher levels in AD children (*p* < 0.001, as illustrated in Table 2). 

### 3.4. PON2/3 Lactonase Activity and Intracellular ROS Levels in PBMNCs 

**Figure 1:**
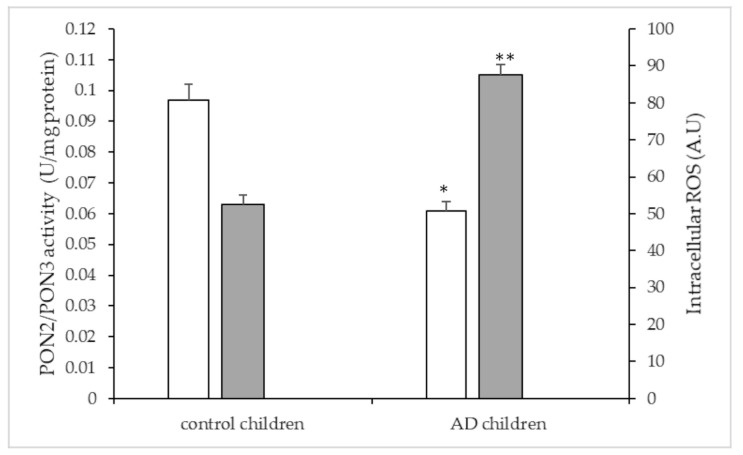
PON2/3 lactonase activity (white bar) and intracellular ROS (grey bar) in peripheral blood mononuclear cells (PBMNCs) isolated from AD and control children. Results reported as mean ± SE. * *p* < 0.001 vs. PON2/3 activity in cells of control children; ** *p* < 0.001 vs. ROS levels in cells of control children.

As reported in Figure 1, mean PON2/3 lactonase activity evaluated in PBMNCs was significantly lower in AD children (0.061 ± 0.002 U/mg) than that in the controls (0.097 ± 0.002 U/mg) (*p* < 0.001). 

The study of intracellular ROS levels in PBMNCs demonstrated that cells isolated from AD children (87.6 ± 2.8 A.U) showed significantly higher ROS levels than those in PBMNCs isolated from controls (52.5 ± 2.6 A.U; *p* < 0.001). 

### 3.5. Correlations

As summarized in Table 3, serum PON1 activities were negatively correlated with serum levels of lipid hydroperoxides, and positively correlated with the total serum antioxidant potential. Significant correlations were established between MPO/PON1 activity and the serum levels of lipid peroxidation and total antioxidant potential.

Positive correlation was observed between serum PON1 activity and intracellular PON2/3 activity. Statistically negative correlation was established between intracellular PON2/3 activity and ROS levels evaluated in PBMNCs (*r* = −0.89, *n* = 104, *p* < 0.001). The activity of PON2/3 in PBMNCs was also negatively correlated with levels of lipid hydroperoxides and MPO/PON1 ratio in serum of subjects (as illustrated in Table 3).

## 4. Discussion

Previous studies reported the involvement of ROS and lipid peroxidation in the pathogenesis of inflammatory skin diseases [19,36,37,38,39]. The relationship between oxidative stress and AD was recently revised [3,4,5]. Higher levels of MDA, a biomarker of lipid peroxidation, were demonstrated [10]. Levels of urinary 8-OHdG were significantly increased in AD patients compared with healthy controls in three studies [5,7,8]. Conversely, other studies reported no significant differences between patients and controls [9].

AD is associated with higher oxidative stress of plasma lipids, as demonstrated by higher levels of serum lipid hydroperoxides and lower values of total antioxidant potential. To investigate the molecular mechanisms potentially involved in higher oxidative stress, we evaluated PON activities and levels of MPO in AD subjects. A decrease in PON1 arylesterase in HDL isolated from serum adult patients affected by AD was previously demonstrated by Trieb et al. [40]. We also observed a decrease in paraoxonase and lactonase activities in serum of AD children. Serum PON1 activity was negatively correlated with serum levels of lipid hydroperoxides and positively correlated with the total serum antioxidant potential. Among HDL-associated enzymes, PON1 exerts a protective effect against the oxidative damage of circulating cells and lipoproteins, and modulates the susceptibility of HDL to lipid peroxidation [22,23,24]. Our results confirmed that higher lipid peroxidation reflects a decrease in PON1 activity. Among factors that contribute to the lipid peroxidation of HDL, the activity of the MPO enzyme could be involved. Undurti et al. showed that, when the enzyme MPO is released in circulation from activated leukocytes, it can bind to HDL, targeting the particle for oxidative modification and functional inactivation [17]. In the present study, MPO levels were significantly higher in the plasma of AD patients, and there was also a significant increase in the ratio between the serum levels of MPO and PON1 activity. The significant increase in the MPO/PON1 (paraoxonase) ratio supports alterations of HDL in AD patients. Other authors demonstrated HDL alterations in AD patients with modifications of HDL lipid and apoprotein composition linked to the formation of dysfunctional HDL and alterations of the interactions of HDL–eosinophils [40,41]. Our findings agree with previous evidence in chronic pediatric dermatitis conditions describing lipid profile alterations [42,43] and a proinflammatory HDL particle with reduced PON1 activity [19].

Alterations in HDL proteins and lipid constituents diminish anti-inflammatory, antioxidant, and endothelial functionalities, and could become proinflammatory [44,45]. 

Previous studies reported functional impairment in PMNs in inflammatory skin diseases [46,47,48]; PMNs from AD patients displayed reduced phagocytic activity intracellular killing [46] and impaired chemotaxis [47]. In our study, using polymorphonuclear cells as an experimental model, for the first time, we demonstrated significantly lower lactonase activity of the PON2/3 enzymes in cells isolated from AD patients. Statistically negative correlation was established between intracellular PON2/3 activity and intracellular ROS levels, suggesting that lower PON2/3 activity could contribute to higher cell oxidative stress. The activity of PON2/3 in PBMNCs was negatively correlated with the levels of lipid hydroperoxides evaluated in serum. Furthermore, positive correlation was observed between serum PON1 activity and intracellular PON2/3 activity in PBMNCs. The activity of PON2/3 was investigated less thoroughly compared to that of PON1; however, the expression of all three PON genes was negatively correlated with numerous inflammatory diseases associated with oxidative stress [21,49]. Previous studies showed that PON2 modulates the formation of intracellular ROS/RNS, and could prevent plasma lipoprotein oxidation [26,27,50]. The suppression of PON2 expression in human vascular endothelial cells is associated with an increase in intracellular ROS formation [26], while the overexpression of PON2 in human vascular endothelial cells, in addition to reducing the formation of intracellular ROS, prevents the lipid peroxidation of LDL induced by incubation with cells [27]. Furthermore, LDL incubated with PON2-def macrophages have a higher level of peroxides than the controls did [50]. Lastly, LDLs isolated from mice overexpressing PON2 are less susceptible to oxidation, and the HDLs of these mice have high antioxidant capacity [50]. These data suggest that intracellular PONs, in concert with the PON1 in HDL, may play a protective role by reducing the production of intracellular ROS and hydroperoxides, and by reducing the oxidation of plasma lipoproteins.

## 5. Conclusions

Our data confirm that AD is associated with higher oxidative damage and a decrease in antioxidant defense. The higher MPO/PON1 ratio in AD serum suggests dysfunctional HDLs in AD. Lower PON activity was also observed in PBMNCs from AD patients. PON2/3 are ubiquitously expressed; therefore, they play a role in reducing intracellular or local oxidative stress. Consequently, in AD patients, alterations of PON activity can promote lipoprotein dysfunctions, carrying out an important role in pediatric atopic dermatitis. Moreover, paired with the lipid profile, MPO and PON measurements could be valuable information to our diagnostic approach and should be included in several clinical studies to evaluate predictive value for cardiovascular events.

Parents of children affected by AD often exhibit fear and anxiety about topical treatments [51]; thus, the recent introduction of nutritional supplements that contain prebiotics and probiotics for prevention and/or being associated with treatments, was viewed with interest [52,53]. On the other hand, the association of oxidative stress in molecular mechanisms involved in the development and maintenance of AD was demonstrated, and it is worthwhile to consider strategies aimed at reducing the oxidative stress of patients. A key role is exerted by lifestyle, dietary habits, and environmental, physical, and psychological factors. A useful approach in daily practice could be to apply personalized diet modifications after a careful evaluation of dietary habits [54], with particular attention given to the intake of fruit and vegetables to provide an appropriate amount of daily vitamins and bioactive molecules.

## Figures and Tables

**Table 1:** Clinical characteristics and plasma parameters in controls and children affected by atopic dermatitis (AD). Results reported as mean ± standard error (SE). TG, triglycerides; TC, total cholesterol; LDL-C, LDL cholesterol; HDL-C, HDL cholesterol; CRP C-reactive protein.

Parameters	Control Children (*n* = 48)	Atopic Children (*n* = 56)
Median age (years)	5.5 ± 3.1	6.1 ± 3.8
Gender (F/M)	26/22	25/31
SCORAD		
<15		17.4%
>15 < 40		82.6%
Asthma		
Presence		18 (32.15%)
Absence		38 (67.8%)
Rhinitis		
Presence		9 (16%)
Absence		47 (83.9%)
TG (mg/dL)	91 ± 11	72 ± 9
TC (mg/dL)	168 ± 6	153 ± 5
LDL-C (mg/dL)	93 ± 4	85 ± 7
HDL-C (mg/dL)	55 ± 5	52 ± 3
ApoAI (mg/dL)	157 ± 11	126 ± 4
HDL-C/ApoAI	0.39 ± 0.03	0.41 ± 0.04
CRP (mg/dL)	0.44 ± 0.18	0.42 ± 0.12

**Table 2:** Serum PON1 activities (paraoxonase, arylesterase, lactonase), lipid hyperoxides, and myeloperoxidase (MPO) levels in controls and AD children; *p* < 0.05 vs. control children. Results reported as mean ± standard error (SE). * *p* < 0.001 vs. control children.

Biochemical Parameters	Control Children	AD Patients
Lipid hydroperoxides (µmol/L)	2.17 ± 0.14	3.58 ± 0.12 *
Total antioxidant potential (mmol TE /L)	13.2 ± 0.21	10.1 ± 0.15 *
PON1—paraoxonase (nmol. min^−1^·mL^−1^)	196.3 ± 8.8	118.7 ± 7.3 *
PON1—arylesterase (U/mL)	77.8 ± 1.9	55.8 ± 1.9 *
PON1—lactonase (U/mL)	31.4 ± 0.5	22.7 ± 0.7 *
MPO levels (ng/mL)	89.6 ± 5.1	128.3 ± 6.1 *
MPO/PON1 (paraoxonase) ratio	0.61 ± 0.04	1.21 ± 0.11 *

**Table 3:** Correlations between biochemical parameters evaluated in serum and PBMNCs isolated from children included in the study (control and AD children, *n* = 104); * *p* < 0.001.

	SERUM				
**SERUM**	PON1 paraoxonase activity (nmol·min^−1^·mL^−1^)		Lipid hydroperoxides (µmol/L)	Total antioxidant potential (mmol TE/L)	MPO/PON1 ratio
	Lipid hydroperoxides (µmol/L)	−0.78 *	-	-	-
	Total antioxidant potential (mmol TE/L)	+0.74 *	+0.77*	-	-
	MPO/PON1 ratio	-	0.76 *	−0.67 *	
**PBMNCs**	PON2/3 activity (U/mg)	+0.79 *	−0.73*	+0.74 *	−0.77 *
	ROS levels (A.U)	−0.69 *	0.76*	−0.62 *	0.76 *

## Data Availability

Data is contained within the article.
